# Efficacy of Repetitive Transcranial Magnetic Stimulation Combined With Visual Scanning Treatment on Cognitive-Behavioral Symptoms of Unilateral Spatial Neglect in Patients With Traumatic Brain Injury: Study Protocol for a Randomized Controlled Trial

**DOI:** 10.3389/fneur.2021.702649

**Published:** 2021-07-14

**Authors:** Francesco Di Gregorio, Fabio La Porta, Giada Lullini, Emanuela Casanova, Valeria Petrone, Loredana Simoncini, Enrico Ferrucci, Roberto Piperno

**Affiliations:** ^1^Azienda Unità Sanitaria Locale, UO di Medicina Riabilitativa e Neuroriabilitazione, Bologna, Italy; ^2^IRCCS, Istituto delle Scienze Neurologiche di Bologna, UO di Medicina Riabilitativa e Neuroriabilitazione, Bologna, Italy

**Keywords:** traumatic brain injury, rehabilitation methods, transcranial magnetic stimulation (repetitive), randomized controlled trial, brain physiology

## Abstract

Left hemispatial neglect (LHSN) is a frequent and disabling condition affecting patients who suffered from traumatic brain injury (TBI). LHSN is a neuropsychological syndrome characterized clinically by difficulties in attending, responding, and consciously representing the right side of space. Despite its frequency, scientific evidence on effective treatments for this condition in TBI patients is still low. According to existing literature, we hypothesize that in TBI, LHSN is caused by an imbalance in inter-hemispheric activity due to hyperactivity of the left hemisphere, as observed in LHSN after right strokes. Thus, by inhibiting this left hyperactivity, repetitive Transcranial Magnetic Stimulation (rTMS) would have a rebalancing effect, reducing LHSN symptoms in TBI patients. We plan to test this hypothesis within a single-blind, randomized SHAM controlled trial in which TBI patients will receive inhibitory i-rTMS followed by cognitive treatment for 15 days. Neurophysiological and clinical measures will be collected before, afterward, and in the follow-up. This study will give the first empirical evidence about the efficacy of a novel approach to treating LHSN in TBI patients.

**Clinical Trial Registration:** https://www.clinicaltrials.gov/ct2/show/NCT04573413?cond=Neglect%2C+Hemispatial&cntry=IT&city=Bologna&draw=2&rank=2, identifier: NCT04573413.

## Introduction

It is estimated that every year in Europe, around 235 persons per 100,000 people are affected by Traumatic Brain Injury (TBI) (1). TBI is a major cause of mortality and morbidity in young people, and its incidence is increasing in persons aged 65 years and older (1). TBI is associated with substantial health care costs, some of which are indirect and long-term, as they are related to loss of productivity and caregiver burden (1). Moreover, TBI is a major cause of long-term disability, impacting patients' and caregivers' quality of life (2).

Left hemispatial neglect (LHSN) is a common condition associated with long-term disability in patients affected by TBI. A recent study showed that about 30% of TBI patients are affected by LHSN (3), a spatial attentive syndrome characterized by a reduced ability to attend, perceive and consciously represent the left contra-lesional space in the absence of a primary sensory deficit (3). Persons with LHSN fail to attend to any stimulus coming from the left-handed space, which can affect the ability to carry out many everyday tasks, such as walking, eating, reading, and getting dressed. Those patients are also often affected by anosognosia for hemiplegia and LHSN. This condition hinders motor and cognitive recovery, predisposes to falling, and reduces independence (3). Furthermore, LHSN in TBI is often associated with a mixture of attention, motor, memory, executive function, and processing speed deficits (1). These impairments lead to a complex cognitive and behavioral picture, which may interfere with standard cognitive treatments (i.e., visual scanning protocols or prism adaptation). Indeed, as scientific evidence for effective TBI treatment is still low, LHSN often remains an untreatable and disabling condition in this population, possibly leading to prolonged length of stay in rehabilitation and a poorer outcome (3).

Rehabilitation methods for LHSN associated symptoms were extensively investigated in persons with right cerebral stroke (4–8). Previous studies showed the efficacy of 1 Hz inhibitory repetitive Transcranial Magnetic Stimulation (i-rTMS) on visuospatial symptoms in persons with an ischemic lesion of the right hemisphere. In particular, i-rTMS was applied to the posterior parietal cortex (PPC) of the unaffected hemisphere for 2 weeks. Remarkably, the observed effects persisted 15 days after i-rTMS treatment (9–11). These results can be explained considering that spatial attention deficit in LHSN due to a right middle cerebral artery territory stroke relates to abnormal activation of the neural system that mediates attentive spatial operations in the healthy brain (12). Lesions of the right PPC (or of the inter-hemispheric connectivity) cause hyperactivity of the left hemisphere. The subsequent inter-hemispheric imbalance leads to a biased attentive allocation toward the ipsilesional space (12). Consequently, inhibition of this hyperactivity may have a rebalancing effect, reducing left spatial attention deficit in LHSN. Moreover, recent studies in stroke patients showed the possibility of improving standard cognitive treatments' efficacy (i.e., visual scanning) if i-rTMS would precede the latter on the unaffected hemisphere (13, 14).

The neural correlates of the inter-hemispheric imbalance associated with LHSN symptoms are often assessed using visual evoked potentials (VEPs). In particular, N1 is a posterior negative deflection in the VEPs, peaking around 180 ms after stimulus presentation, with greater amplitude for stimuli presented in the contralateral hemifield (15). In stroke, it has been demonstrated that LHSN is associated with a smaller amplitude and delayed latency of N1 for left presented stimuli compared to right presented stimuli (15–18). This finding suggests that, in stroke, N1 is a neurophysiological index of impairment on left stimulus processing and, thus, a sign of inter-hemispheric imbalance in LHSN (15, 18, 19). Furthermore, a recent study comparing TBI patients with LHSN against controls showed the presence of hemispheric differences in latencies and amplitudes of the N1 component of VEPs to stimuli presented on both sides (15). These data suggest that the right hemispheric stroke imbalance model could also be applied to explain LHSN symptoms in TBI. In the latter, the hemispheric imbalance could be partly due to a diffuse axonal injury affecting the white matter tracts (20).

What is still unknown is whether in LHSN due to TBI, i-rTMS on the left PPC followed by a visual scanning protocol may be an effective treatment as in right hemisphere stroke, considering that in TBI, the damage is often more widespread and multifocal (1). However, Bonnì et al. demonstrated that a 2-week protocol of i-rTMS (30 Hz Theta burst stimulation on the left PPC) applied to a person affected by LHSN due to TBI reduced the hyper-excitability of the left PPC-primary motor cortex connectivity (20). In this single case, the authors demonstrated a bilateral increase of functional connectivity in the frontal-parietal network on functional Magnetic Resonance Imaging (fMRI). The rebalancing effect induced by brain stimulation was associated with remarkable improvements in LHSN cognitive and behavioral symptoms (20).

According to this preliminary evidence, this study's central hypothesis is that LHSN symptoms in both stroke and TBI rely on similar neurophysiological correlates. Indeed, as observed for right hemisphere strokes, LHSN in TBI patients may be caused by an imbalance in inter-hemispheric activity due to the left hemisphere's hyperactivity (12, 21). Consequently, i-rTMS might have a rebalancing effect by inhibiting this hyperactivity, reducing LHSN symptoms and related disability in TBI patients.

Thus, the general aim of this randomized controlled trial is to test the efficacy of a novel therapeutic approach based on i-rTMS applied to the left PPC followed by a visual scanning treatment (VST) in comparison to the same cognitive treatment preceded by a sham stimulation on neurophysiological and clinical correlates of LHSN in a sample of TBI patients. In particular, this study's specific aims are (1) to assess the efficacy of i-rTMS applied to the left PPC + VST on the inter-hemispheric imbalance in patients affected by LHSN after TBI. In doing so, we will make use of measures of interhemispheric functional connectivity derived from N1. (2) To detail the effect of combined i-rTMS + VST on cognitive symptoms of LHSN in TBI patients as measured by specific clinical measures. (3) To assess whether the effect of the combined i-rTMS + VST treatment has the potential to promote long-lasting lessening of the behavioral manifestations of LHSN in activities of daily living.

## Methods and Analysis

### Study Design

**Figure 1:**
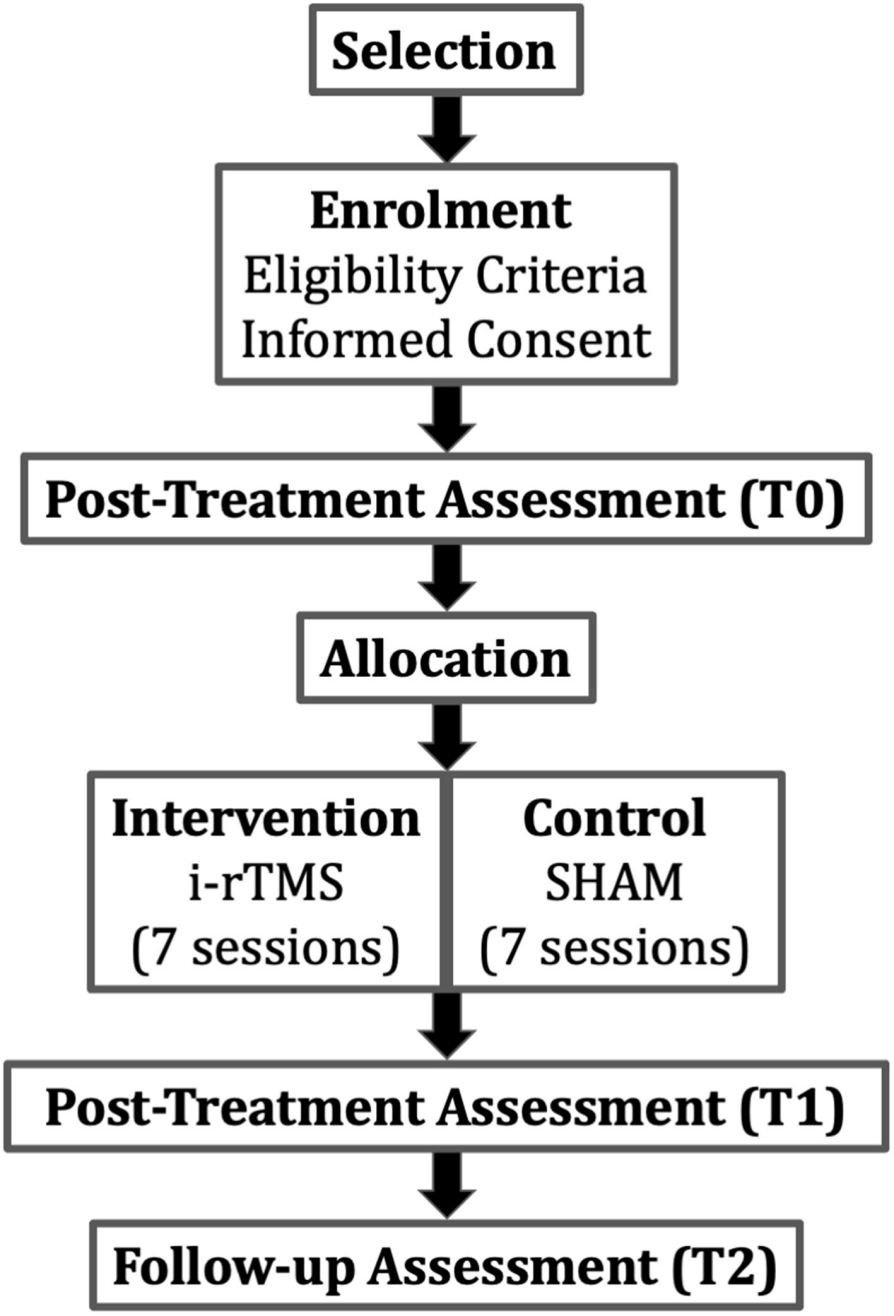
Study flow-chart. The PI or a delegate checks patient's eligibility (i.e., inclusion/exclusion criteria). Selected patients will be assessed (Pre-treatment assessment) before random allocation in the intervention or in the control group. Patients will be assessed also post-treatment and in the 12 weeks follow-up. i-rTMS, repetitive transcranial magnetic stimulation.

The SMaRT TraCE trial (“Stimolazione Magnetica Ripetitiva Transcranica nel Trauma Cranio-Encefalico”; in English: repetitive transcranial magnetic stimulation in traumatic brain injury) is a single-blinded randomized controlled trial (RCT) with pre-test, post-test, and 12 weeks follow-up assessments. The design provides two parallel groups of patients with LHSN symptoms after TBI (i-rTMS + VST and SHAM + VST) with a 2:2 randomized allocation ratio in a superiority trial design. Figure 1 shows the study flowchart.

### Participants

Patients with LHSN due to TBI will be recruited in the Neurorehabilitation Unit of the IRCCS Istituto delle Scienze Neurologiche di Bologna. Subjects will be recruited accordingly to the following eligibility criteria:

Inclusion Criteria:

1. Diagnosis of TBI;
2. Diagnosis of LHSN with specific assessment tests [asymmetry score in the Bells test > 3, (22)];
3. Intra-hospital rehabilitation setting (ordinary hospitalization or DH);
4. Age between 18 and 80 years;
5. Time after injury between 3 weeks and 1 year;
6. Level of cognitive functioning (LCF ≥ 5);
7. Adequate language comprehension to give informed consent. Language comprehension will be considered satisfactory if equal or superior to 75% in ordinary conversation, in the presence of an aphasic disturbance or deafness. Use of eventual hearing aids is allowed. In case of doubts, a simple or language comprehension test (token test) will be administered.
8. Presence of inter-hemispheric asymmetries in the EEG activity evidenced by qualitative evaluation;

Exclusion Criteria:

1. Medical instability at enrollment, defined as the acute onset of an unexplained derangement of vital parameters (i.e., temperature, blood pressure, pulse rate, respiratory rate, oxygen saturation, level of responsiveness) outside the normal range (for example, fever, acute internist conditions, etc.) and/or the onset of any new medical condition requiring unexpected additional diagnostic procedures and treatments (for example, severe pain, reduction of urinary output, etc.);
2. Presence of epileptogenic alterations to the EEG and/or previous epileptic seizures;
3. Presence of intracranial implants of a metallic material;
4. Presence of devices that could be altered by i-rTMS, such as pacemakers, ventriculoperitoneal shunt, baclofen pump;
5. Decompressive craniectomy;
6. Drugs conditioning the state of consciousness-vigilance such as benzodiazepines;
7. Cortical blindness and/or visual agnosia;
8. Concomitant psychiatric disorders and/or history of substance abuse;
9. Post-traumatic agitation;
10. Post-traumatic complications (i.e., hydrocephalus).

The principal investigator (PI) or a delegate will check the eligibility criteria before enrollment. After verifying the eligibility criteria, the PI will provide eligible patients and their caregivers with all the information and details relative to the study in simple language.

### Intervention

Intervention is based on previous studies (4, 9, 10) and on an RCT protocol for LHSN after stroke (13). In particular, seven sessions of i-rTMS will be administered over 15 days (9). In detail, the parameters used in each session will be:

1. The stimulation coil will be positioned tangentially over P5 accordingly to the international EEG 10/20 system, which corresponds to the target non-lesioned left posterior parietal cortex (4);
2. 90% of the motor threshold;
3. Frequency: 1 Hz;
4. Each session consisted of one train of 900 pulses, which resulted in a whole stimulation period of 15 min.

The stimulation site was chosen based on previous studies. In those studies, TMS over P5/P6 (9, 23) or P3/P4 (24, 25) was shown to reduce contralesional neglect related symptoms in patients with a unilateral brain lesion.

Each i-rTMS session will last 15 min and be administered every other day (e.g., Monday-Wednesday-Friday, Monday-Wednesday-Friday, Monday).

VST is a conventional cognitive protocol based on the administration of a structured series of tasks aiming at improving spatial exploration abilities (26). It provides various visual scanning tasks to increase the patient's awareness of the LHSN clinical manifestations and teach strategies to improve spatial exploration abilities (10). VST will be administered following the i-rTMS. In particular, three different training tasks will be used:

1. Visuospatial training;
2. Reading and copying training;
3. Copying of line drawings on a dot matrix.

All training tasks include three increasing levels of difficulty, thus giving nine possible task-difficulty combinations. Each level of difficulty will be practiced until the subject will reach a level of accuracy of 75%.

The training will be carried out in 50 min sessions for 5 days a week within 15 days (10) for 11 sessions. When the i-rTMS is also carried out, the visual scanning protocol administration will immediately follow the brain stimulation.

### Control

In the control group, a SHAM placebo stimulation is implemented. SHAM stimulation parameters are the same as the intervention stimulation, but the TMS coil will be positioned at 90° on the target area. Thus, no specific cortical modulation will be implemented (SHAM stimulation). The VST protocol will be administered with the same modalities and time frame for this group, as detailed for the intervention group. All routine care is permitted for both groups during the study period.

### Outcomes

Our operational objectives and outcome measures are divided into primary and secondary endpoints to reach all aims. Our outcomes will allow us to evaluate different clinical and neurophysiological aspects of LHSN and any differential improvements induced by the rehabilitation protocol.

#### Primary Endpoint

The primary outcome is represented by a specific assessment of inter-hemispheric imbalance with connectivity indexes derived from N1 amplitude (i.e., vABI Visuospatial Attention Bias Index) (13) and latency (i.e., IHTT, Inter-Hemispheric Transmission Time) (27–29).

Visual evoked potentials (VEPs) and lateralized visual processing will be collected accordingly to a method already introduced for LHSN assessment in stroke patients (13, 17, 21).

**Figure 2:**
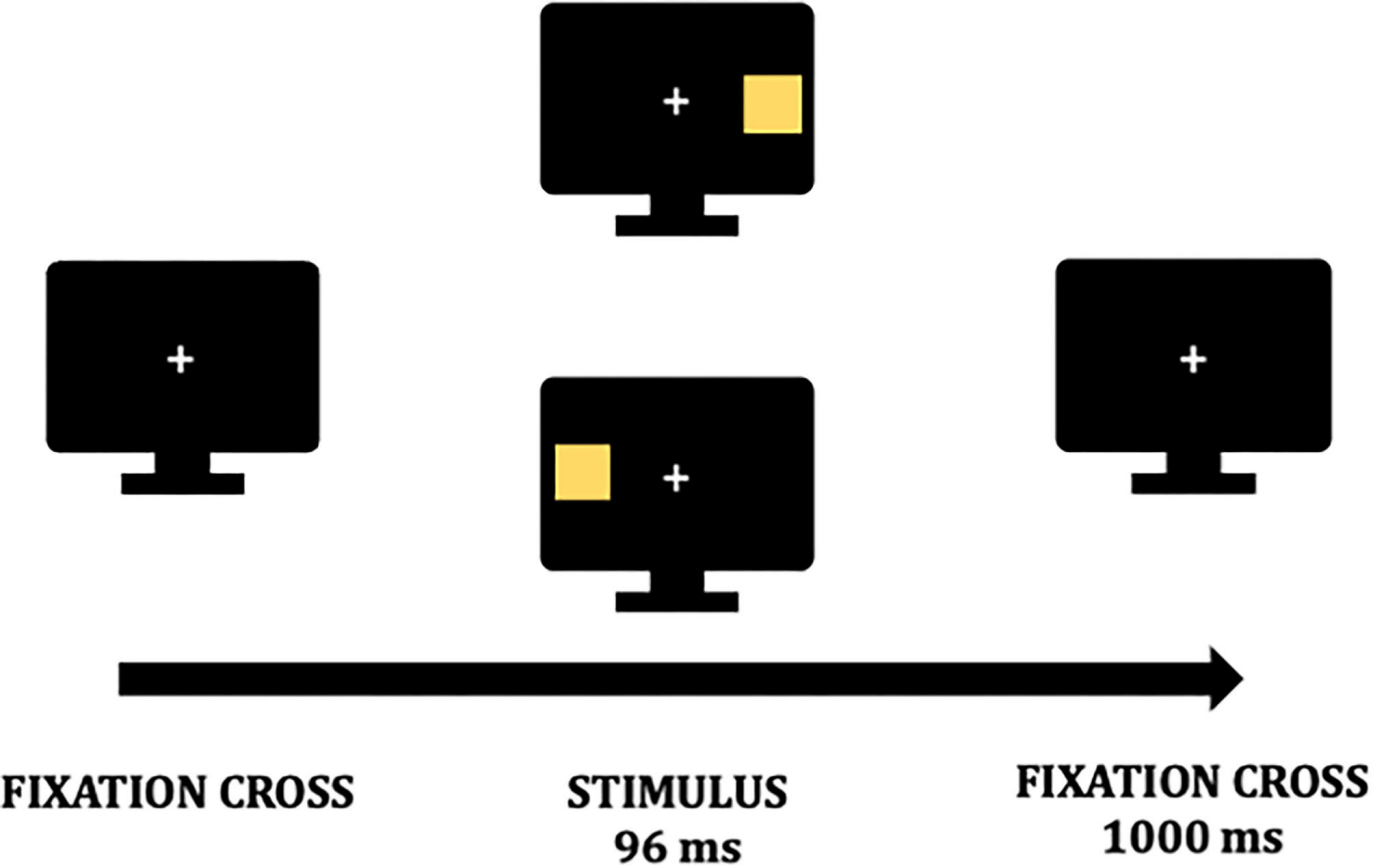
Visual detection task. A fixation cross is presented on a black background during the whole task. At the beginning of a trial, a stimulus is presented randomly for 96 milliseconds (ms) either on the fixation point's left or right. Before a new trial begins the fixation cross is again presented for 1,000 ms.

In particular, VEPs are collected during a passive visual detection task with lateralized stimuli (13, 18) to investigate inter-hemispheric imbalance in LHSN (12, 30, 31). As reported in a previous study (13), the EEG will be recorded while patients perform the task on a computer screen. The view distance will be 50 cm from the screen. A white central fixation cross is displayed on a black background (Figure 2). Patients are instructed to fix the cross during the whole task, and whenever participants lose the fixation, feedback will be provided to recover it. A stimulus will be presented randomly on the fixation cross' either sides at a distance angle of 28° along the midline on each trial. Stimuli are 1 × 1 cm yellow squares and will be displayed for 96 ms. Before the subsequent trial, a black background with the fixation cross will be presented for 1,000 ms (Figure 2). Four blocks of 64 stimuli will be delivered, and the overall task will have an average duration of 20 min.

The EEG will be recorded from 18 Ag/AgCl-cup electrodes according to the 10/20 system referenced to the linked ear lobes. The EEG signal will be recorded from electrodes: Fz, Cz, Pz, C4, C3, P4, P3, F4, F3, Oz, O1, O2. The negative peak of the N1 will be considered to analyze amplitude in microvolt (μv) and latency in milliseconds (ms). N1 amplitude and latency will be analyzed separately for left and right-presented stimuli over P3 and P4 (13, 17). Then, indexes of inter-hemispheric imbalance will be extracted. In particular, the Visuospatial Attention Bias Index (vABI; 13) and the Interhemispheric transmission time [IHTT, (27)], which are based, respectively, on N1 amplitude and latency.

The vABI will be extracted in two steps. First, we will calculate the averaged activities of the N1 for stimuli presented on both sides from considered electrodes (i.e., mean of P3 and P4). These averaged activities will measure the two hemispheric activations after lateralized stimulus presentation. The individual differences in the activations for lateralized stimuli will be calculated according to the following formula: vABI = N1 amplitude for right stimuli - N1 amplitude for left stimuli. The vABI will be calculated as a lateralization index (32) on N1 amplitude. Such an index can measure imbalance for left and right stimuli processing as it measures the activation of the two hemispheres in response to lateralized stimuli.

Similarly, based on N1 peak latency, we will compute the IHTT (27, 28), an indicator of the EEG signal's transmission times from one hemisphere to the other. In particular, IHTT measures the difference between N1 latencies for left presented stimuli on the posterior electrodes P3 and P4 (i.e., left IHTT = N1 latency on P4 - N1 latency on P3), thus constituting a single index in milliseconds of the inter-hemispheric transmission times specifically for left presented stimuli (13).

#### Secondary Endpoints

The secondary endpoints focus on the impact of rTMS on clinical and motor indexes. In particular, we will test visual-spatial attentive functioning with a standard neuropsychological battery for LHSN assessment, the Behavioral Inattention Test [BIT; (33)]. The BIT consists of two subscales (cognitive and behavioral) with standardized scores, where lower ratings indicate a more severe visual-spatial impairment. The degree of functional independence in daily living activity (i.e., eating or reading) will be tested with the Catherine Bergegò Scale (CBS). In contrast, we will test motor ability with the Motricity Index (MI) Trunk Control Test (TCT) and the motor subscale of the Functional Independence Measure (FIM^tm^) (34, 35). Finally, attentive functioning will be tested with a specific subtest (i.e., alertness and Visual Field/Neglect) of the Test of Attention Performance (TAP), an attentive computerized battery (13, 35).

## Data Analysis

### Sample Size

The sample size was calculated using the following formula:

$$\begin{array}{l} n=\frac{2{\left(z_{\left(1-\alpha \right)}\;+z_{\left(1-\beta \right)}\right)}^{2\;}\sigma^{2}}{\delta^{2}} \end{array}$$

Where *N* is the sample size; σ2: population variance established by previous studies = 2.1; δ2: absolute error allowed for parameter estimation for vABI = 2.8; z: constant (corresponding to the value of the standardized normal random variable) that depends on the level of confidence desired for the estimation. Fixing α = 0.05 and 1 – β = 0.80, (Z_1−α_ + Z_1−β_) ^2^ = 10.5. The sample size resulting from the formula is 24. Consequently, the minimum sufficient sample to reach all aims is, assuming 10% of subjects lost to follow-up, 28 subjects (14 X group), enrolled over 1 years. In case of recruitment delays, a multicentric extension of the study will be considered to guarantee an adequate number of participants.

### Statistical Analysis

Differences in vABI and IHTT will be analyzed between the pre-treatment (T0), post-treatment (T1), and follow-up (T2) phases for both groups of patients (group r -TMS + TCC and SHAM + TCC group) to evaluate neurophysiological correlates of LHSN in the two groups.

In randomized controlled trials, the recommended statistical procedure to test pre-, post-treatment, follow-up control group design is the analysis of covariance (ANCOVA) (36, 37). In the context of ANCOVA, post-treatment and follow-up are considered dependent variables with pre-treatment variables as covariate (36, 37). However, ANCOVA requires the satisfaction of two assumptions, i.e., homogeneity vs. heterogeneity of the population at the pre-treatment time point and normal data distribution. In the present study, we will verify first the two ANCOVA assumptions for each outcome measure and, afterward, we will adopt the appropriate corrections and statistical approaches as follows:

1. Homogeneity vs. heterogeneity test: For each outcome, homogeneity of regression slopes will be verified (38): i.e., the values of *b* should not be significantly different between groups. If the assumption is violated, a CHANGE measure (i.e., a score of gain in the specific outcome) will be considered in a two × two mixed-model ANOVA with the between factor group and the within factor time (post-treatment and follow-up).
2. Normal distribution test: Normal distribution will be verified (i.e., Kolmogorov-Smirnov test larger than 0.05) (36, 37) for each outcome variable. In case the assumption is violated, a linear logistic transformation will be performed.

If ANCOVA assumptions are verified for the primary outcome, we will analyze covariance for each neurophysiological index using a mixed-model ANCOVA with a 2X3 design. The “between” factor will be represented by the randomization group (rTMS, SHAM), whereas the “within” factor will be the assessment time (T1, T2) with T0 as a covariate. Whenever necessary, Greenhouse-Geiser correction will be applied, and corrected *p*-values will be reported in the ANCOVA. Further adjustments will be made for other possible confounding factors such as age, gender, education of the participants, and lesion location (if heterogeneity between participants in lesion locations emerges from clinical data). Besides *p*-values, effect sizes will be provided to assess the size of the treatment effect. Similar analyses will also be performed for clinical and motor outcomes after verification of ANCOVA assumptions. The BIT, the TEA, the CBS, and the motor function tests provide standard scores, separated for each test, and scores including a general performance with cut-offs that allow discriminating pathological performance. Several mixed-model ANCOVAs will be applied for every test in the 2X3 design described above. Finally, we will perform correlation analyses (both parametric and non-parametric) to evaluate the relations between the neurophysiological indices and clinical measures.

Two-tailed *t*-tests for independent samples will be employed to investigate the differences between groups. Data analysis will be performed using MatLab (The Mathworks Inc.) and SPSS (version 13). A significance level of 5% (i.e., *p*-value = 0.05), corrected for multiple comparisons when needed, will be accepted.

Additional clinical and demographic information, such as handedness of subjects, prescribed medications, and relevant medical conditions, will be recorded for each participant in specific Case Report Forms.

## Specific Procedures

### Minimizing Inter-Rater Measurement Bias

The medical doctor who will administer the stimulation will always be the same; however, assessors will change between pre and post-measurements. To minimize biases deriving from inter-rater measurement errors, the following interventions will be performed before the start of the trial:

1. Collegial assessments to standardize administration modalities and scoring procedures.
2. Development of an “assessment manual” containing all information for administering and scoring procedures.

### Assignment of Interventions and Data Management

#### Allocation

Participants will be allocated randomly in the active rTMS group or the SHAM placebo group. A blocked randomization list (2:2 per group) will be generated using the online software QuickCalcs (www.graphpad.com). Only the PI and the physician administering i-rTMS will have access to the randomization list.

#### Blinding

To ensure a double-blind assessment in all phases (T0 pre-treatment, T1 post-treatment, T2 follow-up), assessors will not be aware of the patient's randomization group. Moreover, pre-treatment assessments (T0) will be performed before randomization. Also, the visual scanning protocol will be administered by therapists unaware of the patient's allocation. Patients themselves will be instructed not to reveal any information about the brain stimulation treatment received.

#### Data Collection and Management

All data will be anonymized, and a specific alpha-numeric code will be attributed to each subject after enrolment. An electronic study database will be configured with all patient details, including the randomization group.

### Safety Assessment

Recently, guidelines for stimulation protocols were defined (39). Therapeutic interventions in clinical context should have the following properties:

- The application should be easy to implement without neuroimaging and neuronavigation systems to localize the target area. Many studies, as an alternative to neuronavigation, adopt the international 10/20 localization system.
- The total application time of the daily rehabilitation paradigm should not exceed ten sessions during 2 weeks. Protocols that provide daily applications for more than 2 weeks are difficult to implement in rehabilitation centers and may not be tolerated by patients.

rTMS, when administered according to the international guidelines, is a safe technique.

The stimulation paradigm (9, 13, 14) reaches the guidelines mentioned above for brain stimulation protocols (39). Moreover, we followed a TMS methodological checklist (40) to report methodological details about the stimulation protocol and thus improve the quality of data collection and replicability of the study. However, adverse events are reported in the literature, as follows:

- Local annoyance in the stimulated area (frequent): this effect rarely requires the suspension of rTMS.
- Headaches (quite frequent but usually mild). We will administer analgesics (e.g., paracetamol) in case of annoying headaches.
- Temporary loss of hearing (rare) for the duration of the stimulation session.
- Epileptic (fairly rare) crises, which occur in predisposed individuals. To minimize this risk, subjects who have suffered from seizures during the acute phase or have a diagnosis of epilepsy will be excluded from the trial (exclusion criterion).

Any adverse events during treatment will be recorded in a specific Case Report Form (CRF) and reported at the end of the study. Also, the physician who will administer i-rTMS will manage any adverse events occurring during the administration. Should the treatment be suspended, the reason will be reported. Data will be analyzed accordingly to the “intention to treat” principle and included in the study's final report.

### Roles and Responsibilities

Patients will be enrolled within the Physical Medicine and Neuro-rehabilitation Unit of IRCCS Istituto delle Scienze Neurologiche di Bologna (i.e., the coordinating center).

### Oversight and Monitoring

A designated external committee will perform data monitoring, database, and statistical analysis management. Statistical analyses over the complete dataset will be performed at the end of data collection. However, interim analyses approved by the local ethical committee may be performed. The PI will coordinate clinical trial's organizational, ethical, and scientific aspects. Only the PI can declare the end of the enrolment.

## Discussion

Many published studies have highlighted the efficacy of different rehabilitation methods for LHSN syndrome after stroke (10). However, scientific evidence is still low in TBI patients, given factors such as low sample size, methodological bias (lack of double-blind studies or follow-up assessments), and contradictory results.

This study's rationale is that neuronal loss due to TBI leads to an impairment of cognitive functions due to a deficit in the related neuro-functional networks. Thus, the reactivation of those networks may allow the empowerment of the compromised functions. Unlike the traditional rehabilitation methods, the new magnetic stimulation techniques, such as i-rTMS, allow the execution of a cognitive task through the pre-empowerment of a specific network or neuronal circuit. This priming may facilitate experiential learning with a richer and more articulated neural environment and selectively stimulate according to the areas most involved in the lesion. In TBI, the mechanism of empowerment concerns the preserved areas and the inter-neuron connectivity between those areas. Consequently, i-rTMS could increase the “responsiveness” of the peri-lesional areas and the inter-hemispheric connectivity during cognitive training, increasing its effectiveness compared to the SHAM condition.

Therefore, the current project's main expected outcome will provide evidence, on a large sample of TBI patients, of the inter-hemispheric functionality underlying cognitive symptoms of LHSN. It will also point out the specific effect of i-rTMS protocols on the inter-hemispheric imbalance. In particular, we expect to observe in the treatment group a larger rebalancing effect than in the control group, as demonstrated by smaller amplitudes of vABI and earlier latencies of IHTTs at the post-treatment assessment. Furthermore, we expect to observe the persistence of this effect at follow-up. Additionally, we expect to observe larger improvements in cognitive and behavioral symptoms of LHSN induced by the i-rTMS compared to the control group, as demonstrated by better performances on clinical tests and batteries (10).

To our knowledge, this study will be the first to provide an evidence-based theory on the inter-hemispheric functionality in TBI patients with LHSN, providing clinicians with a new framework for approaching, studying, and interpreting LHSN with innovative markers on neurophysiological activity. Moreover, the evaluation of rehabilitation effects of i-rTMS on the visuospatial inter-hemispheric network and cognitive and activities of daily living measures of LHSN will provide the basis to understand how i-rTMS influences LHSN in TBI patients. This novel knowledge, in turn, will create the basis for the development of new treatment strategies, which will have the potential to lessen the impact of LHSN-related disability for TBI patients and their families.

## Conclusions

The SMART-TRACE is a protocol for the rehabilitation of attentive spatial deficits in TBI patients, based on therapeutic approaches already established for stroke patients, which combines brain stimulation and cognitive treatments. The current protocol is easily applicable and relatively low-cost. Although a TMS stimulator is necessary for the intervention procedure, the visual scanning protocol is very flexible regarding the materials needed and the cognitive tasks. Flexibility is indeed a crucial aspect, considering the clinical heterogeneity of TBI patients. For instance, visual scanning can be implemented at the bedside, and also patients with severe motor impairments can easily carry out the tasks. Should the efficacy of the study protocol be demonstrated, it could be implemented in ordinary clinical practice, thus providing a valuable therapeutic option to reduce LHSN related symptoms and improves the clinical outcome in TBI patients.

## Trial Status

The protocol here presented was registered in October 2020 on ClinicalTrials.gov (NCT04573413; title: “Repetitive Transcranial Magnetic Stimulation in Traumatic Brain Injury”). The estimated study completion date is March 2023.

## Ethics Statement

The studies involving human participants were reviewed and approved by Comitato Etico Indipendente di Area Vasta Emilia Centro (CE-AVEC; CE n.19062). The patients/participants provided their written informed consent to participate in this study.

## Author Contributions

FD is the study's principal investigator and coordinates organizational, ethical, and scientific aspects of the clinical trial. EC is responsible for the TMS stimulation. FD, EC, FL, and RP provided the idea and designed the protocol. VP, LS, and EF are responsible for cognitive, motor, and neurophysiological assessments. FD, FL, and GL wrote the manuscript. All authors have read and approved the final version of the paper.

## Conflict of Interest

The authors declare that the research was conducted in the absence of any commercial or financial relationships that could be construed as a potential conflict of interest.
